# Development of an *In Vivo* RNAi Protocol to Investigate Gene Function in the Filarial Nematode, *Brugia malayi*

**DOI:** 10.1371/journal.ppat.1001239

**Published:** 2010-12-23

**Authors:** Chuanzhe Song, Jack M. Gallup, Tim A. Day, Lyric C. Bartholomay, Michael J. Kimber

**Affiliations:** 1 Department of Biomedical Sciences, College of Veterinary Medicine, Iowa State University, Ames, Iowa, United States of America; 2 Department of Veterinary Pathology, College of Veterinary Medicine, Iowa State University, Ames, Iowa, United States of America; 3 Department of Entomology, College of Agriculture and Life Sciences, Iowa State University, Ames, Iowa, United States of America; Stanford University, United States of America

## Abstract

Our ability to control diseases caused by parasitic nematodes is constrained by a limited portfolio of effective drugs and a paucity of robust tools to investigate parasitic nematode biology. RNA interference (RNAi) is a reverse-genetics tool with great potential to identify novel drug targets and interrogate parasite gene function, but present RNAi protocols for parasitic nematodes, which remove the parasite from the host and execute RNAi *in vitro*, are unreliable and inconsistent. We have established an alternative *in vivo* RNAi protocol targeting the filarial nematode *Brugia malayi* as it develops in an intermediate host, the mosquito *Aedes aegypti*. Injection of worm-derived short interfering RNA (siRNA) and double stranded RNA (dsRNA) into parasitized mosquitoes elicits suppression of *B. malayi* target gene transcript abundance in a concentration-dependent fashion. The suppression of this gene, a cathepsin L-like cysteine protease (*Bm-cpl-1*) is specific and profound, both injection of siRNA and dsRNA reduce transcript abundance by 83%. *In vivo Bm-cpl-1* suppression results in multiple aberrant phenotypes; worm motility is inhibited by up to 69% and parasites exhibit slow-moving, kinked and partial-paralysis postures. *Bm-cpl-1* suppression also retards worm growth by 48%. *Bm-cpl-1* suppression ultimately prevents parasite development within the mosquito and effectively abolishes transmission potential because parasites do not migrate to the head and proboscis. Finally, Bm-cpl-1 suppression decreases parasite burden and increases mosquito survival. This is the first demonstration of *in vivo* RNAi in animal parasitic nematodes and results indicate this protocol is more effective than existing *in vitro* RNAi methods. The potential of this new protocol to investigate parasitic nematode biology and to identify and validate novel anthelmintic drug targets is discussed.

## Author Summary

Lymphatic filariasis is a debilitating tropical disease caused by parasitic nematodes such as *Brugia malayi* that are transmitted to humans through the bite of infected mosquitoes. Controlling lymphatic filariasis, and other parasitic nematode diseases, is made difficult by a limited repertoire of sub-optimal drugs and substantial experimental roadblocks to new drug development. We have developed a novel and highly effective *in vivo* RNA interference (RNAi) methodology to better understand gene function in the parasitic nematode, *B. malayi*. RNAi triggers are delivered to developing parasites in the mosquito intermediate host – an improvement on existing RNAi protocols, in which the parasite is removed from the host and maintained in liquid culture. Using this protocol we have significantly suppressed a cathepsin gene in *B. malayi*, which resulted in dramatic change to parasite movement, growth and development in the mosquito to the extent that worms cannot be transmitted to a new host. These data underscore the power of this protocol to 1) critically assess gene function in parasitic nematode biology, 2) to reveal the mechanism of action for existing drugs, and 3) for discovery of novel drug targets for drug development.

## Introduction

Lymphatic filariasis is a disease caused by filarial nematodes including *Wuchereria bancrofti* and *Brugia malayi*, transmitted through the bite of infected mosquitoes. These parasites perpetuate socioeconomic instability in developing countries by inflicting crippling morbidity and debilitating stigmatization. The impact of this disease is vast - over 120 million people are infected in 81 endemic countries [1]. In an effort to alleviate morbidity and eliminate transmission of this disease, the Global Program for the Elimination of Lymphatic Filariasis (GPELF) has orchestrated a systematic mass drug administration (MDA) program centered on the repeated dosing of either diethylcarbamazine citrate (DEC) and albendazole or albendazole and ivermectin in areas where the other filarial parasites, *Onchocerca volvulus* and *Loa loa* are co-endemic. This strategy has reduced prevalence in many areas [2] but lymphatic filariasis remains a significant global health concern. Many factors contribute to continued transmission, but central is the inadequate portfolio of effective drugs; none of the MDA drugs are effective against all life stages of the parasite with notable inefficacy against adult worms [3]–[5]. This means MDA must be provided annually for the duration of the lifespan of adult parasites. This situation is compounded by gaps in our understanding of mechanisms of drug action and pharmacology – the site of action of DEC is unknown despite being the drug of choice for lymphatic filariasis control for decades, and the filaricidal mechanism of ivermectin at therapeutic concentrations is also equivocal. There is a very real and significant need for additional and more effective antifilarial drugs, and a better understanding of the mode of action of existing drugs [6].

A major obstacle to the rational development of such drugs is the experimental intractability of parasitic nematodes. An example of this complication is RNA interference (RNAi), a reverse genetic tool that allows researchers to rapidly and specifically ‘turn off’ genes of interest. RNAi has fast become a standard tool in rational drug discovery for the identification and validation of potential new drug targets [7], [8]. By suppressing specific genes and examining the resulting phenotype, it is possible to delineate gene function and appraise the potential value of encoded proteins as drug targets. Successful applications of present RNAi protocols to parasitic nematodes have been sporadically reported, limited in their effectiveness and seldom repeated [9]. Some success has been achieved with *Nippostrongylus brasiliensis* [10], *Ascaris suum* [11], *Trichostrongylus colubriformis* [12], *Ostertagia ostertagi* [13] and *Haemonchus contortus* [14], [15]. Germane to the study of filarial worms, RNAi has been described in *B. malayi* [16], [17], *Onchocerca volvulus* [18], [19] and *Litomosoides sigmodontis* [20]. The conclusion has been reached, however, that successful RNAi “only works on a limited number of genes, and in some cases the effect is small and difficult to reproduce” [14]. The inability to depend on present RNAi protocols with parasitic nematodes has proved a major stumbling block to the identification and validation of new drug targets, to a better understanding of anthelmintic mode of action, and to advancing our comprehension of parasite biology.

The recalcitrance of animal parasitic nematodes to RNAi is perplexing, given that *Caenorhabditis elegans*, a free-living nematode, and plant parasitic nematodes are readily susceptible to the technique [21]–[28]. One hypothesis advanced to explain this recalcitrance is that because present RNAi protocols employ *in vitro* approaches including soaking nematodes in an RNAi trigger, feeding nematodes bacteria producing the trigger, or electroporating of the trigger into the parasite, the RNAi trigger is not provided in a manner conducive to systemic gene suppression [29]. Implicit in the use of these protocols is the removal of a parasite from the host and its maintenance in a liquid culture. Therefore these protocols have distinct drawbacks such as difficulty maintaining healthy, viable worms that behave normally *in vitro*, limitation of use of parasites or life stages for which *in vitro* culture is defined, and poor efficacy in RNAi trigger delivery methods that can prove lethal to the parasite [30].

The aim of this study was to develop an innovative *in vivo* approach to RNAi in parasitic nematodes that overcomes the drawbacks associated with present *in vitro* experimental paradigms. Our approach is based on the filarial nematode *B. malayi*. We establish a *B. malayi* infection in an intermediate host, the mosquito *Aedes aegypti*, and then initiate suppression of parasite genes by injecting an RNAi trigger directly into the mosquito. The mosquito acts as an ideal culture and delivery system, ensuring the RNAi trigger is exposed to healthy, developing parasites. Using this approach we have effectively and quantifiably suppressed expression of *Bm-cpl-1*, a *B. malayi* gene encoding a cathepsin L-like cysteine protease. Dramatic aberrant phenotypes accompany this suppression, including a marked retardation of motility, an inhibition of normal parasite migration behavior within the mosquito and impaired parasite growth and development. Suppression is specific; non-target RNAi has no effect on nematode viability or behavior, and the level of gene suppression and extent of the resultant phenotypes suggest this new protocol is more effective than previous methods. The development of an *in vivo* RNAi protocol to reliably suppress gene expression in filarial worms has great potential for the identification and validation of novel drug targets, and more broadly, to explore parasitic nematode biology and host-parasite interactions.

## Results

### A *Brugia* RNAi trigger rapidly disseminates throughout the mosquito host

**Figure 1:**
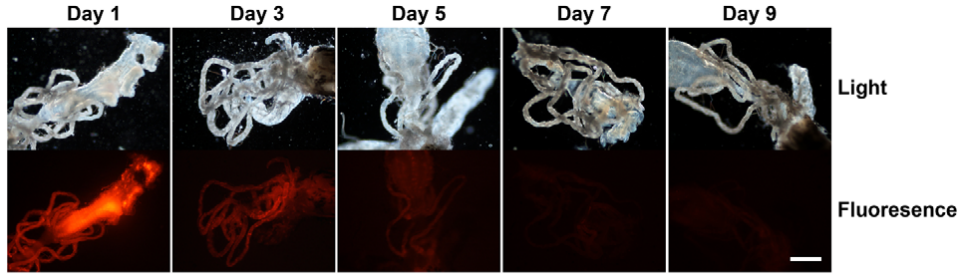
Dissemination and persistence of intrathoracically injected Cy 3-labelled *Brugia malayi* Cathepsin-L1 siRNAs in *Aedes aegypti*. Midgut and Malpighian tubule tissues are shown in light (upper panel) and fluorescence (lower panel) micrographs from 1 to 9 days post-injection (scale bar 100 µm).

Our hypothesis is that mosquitoes provide an optimal culture and delivery system for an RNAi trigger targeted to developing *Brugia malayi* parasites. Healthy, viable, developing parasites are subjected to the RNAi trigger because the parasites undergo growth and development in the mosquito intermediate host. In order to test the extent of dissemination of the RNAi trigger from the site of intrathoracic injection, 150 ng of an equimolar mix of four 3′ Cy 3-labelled *Bm-cpl-1* siRNAs was injected into adult *Aedes aegypti* mosquitoes as described. The dissemination of this RNAi trigger through the mosquito was tracked over 15 d post-injection by periodic microdissection of the mosquito and evaluation of internal fluorescence compared to saline injected controls. The labeled siRNA mix spread rapidly from the site of injection and maximal fluorescence was observed 24 h post-injection (Fig. 1). The intensity of fluorescence slowly decreased until reaching basal levels at five d post-injection after which fluorescence intensity was not appreciably different from control mosquitoes. Our observations closely parallel those of a previous report that describes the spread of 140 ng AlexaFluor 555-labeled siRNA in the mosquito *Anopheles gambiae* from an injection site to the midgut and pericardial cells 36 h post-injection [31]. Systemic dispersion and persistence of RNAi signal from the site of injection suggests *B. malayi* larvae are likely to be exposed to the RNAi trigger in our experimental model.

### *Brugia* gene suppression *in vivo* is potent and specific

**Figure 2:**
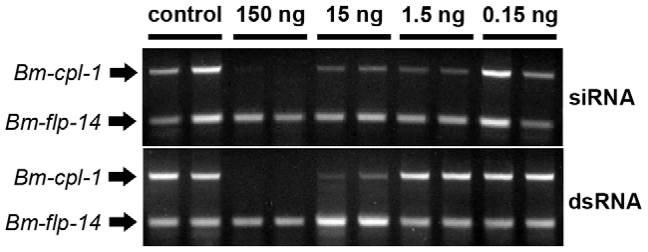
Concentration-dependent, *in vivo* suppression of *Brugia malayi* Cathepsin-L1 (*Bm-cpl-1*) using siRNA (Top) or dsRNA (Bottom) RNAi triggers. Micrograph shows ethidium bromide stained agarose gel electrophoresis of relative RT-PCR analysis of individual, *B. malayi*-infected mosquitoes 48 h post-injection of RNAi trigger at 10 d post-infection. Amplified product for the target gene, *Bm*-*cpl-1*, is shown above a neuropeptide reference gene (*Bm-flp-14*).

Recently it has been shown that *B. malayi* genes encoding cathepsin L-like enzymes can be suppressed *in vitro* by soaking adult parasites in culture media containing siRNA [17]. We tested the capacity of our novel methodology to suppress larval stage *B. malayi* gene expression *in vivo* by injecting mixed siRNAs specific to the cathepsin L-like *Bm-cpl-1* gene directly into *Ae. aegypti* mosquitoes harboring L3 stage *B. malayi* parasites. Gene suppression was assayed 48 h post-injection using a semi-quantitative RT-PCR in which the intensity of *Bm-cpl-1* amplification in the linear phase of the reaction was compared to an internal *B. malayi* reference gene (*Bm-flp-14*) that is expressed stably and at comparable levels to *Bm-cpl-1*. Control mosquitoes were injected with equal volumes of *Aedes* physiologic saline. This methodology was optimized to amplify *Bm-cpl-1* from a heterogeneous mosquito/parasite total RNA preparation from a single mosquito. Suppression was concentration-dependent because injection of 0.15 ng siRNA did not appear to reduce *Bm-cpl-1* transcript levels. However, injection of 15 ng or 1.5 ng of siRNA decreased transcript levels, and injection of 150 ng mixed siRNA into mosquitoes profoundly suppressed *Bm-cpl-1* expression; the target parasite gene could not be amplified (Fig. 2). This suppression was also specific; expression of the *Bm-flp-14* reference gene was unaffected by siRNA injection and target gene expression was normal in saline-injected controls.

Application of dsRNA is the commonly used method for triggering RNAi in parasitic nematodes and has advantages over siRNA; dsRNA can be generated in-house more quickly than commercially produced siRNAs at lower cost. *B. malayi*-infected mosquitoes were also subjected to treatment with dsRNA as an RNAi trigger. The effect of dsRNA was concentration-dependent such that injection of 15 ng dsRNA results in *Bm-cpl-1* suppression but 1.5 ng dsRNA had no appreciable effect. Injection of 150 ng of dsRNA potently suppressed *Bm-cpl-1* transcript abundance and suppression appeared specific, with *Bm-flp-14* expression unaffected by dsRNA (Fig. 2).

**Figure 3:**
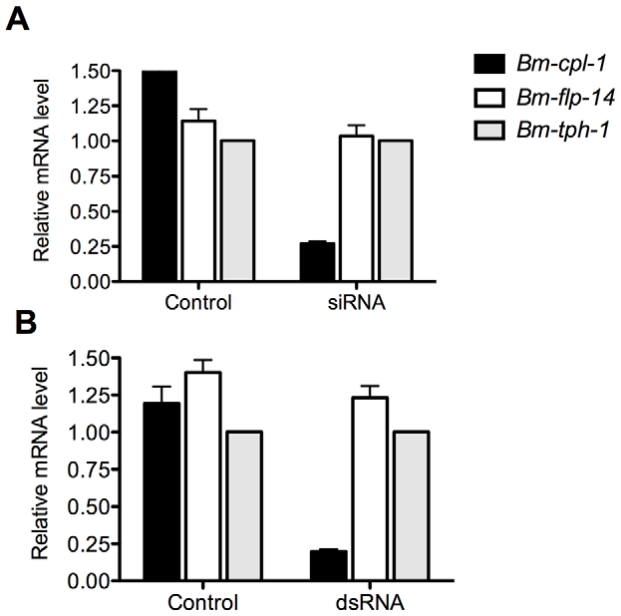
Quantitative PCR demonstrates significant reduction in *Bm-cpl-1* transcript levels as a result of siRNA and dsRNA RNAi trigger injection into *B. malayi*-infected *Ae. aegypti*. Both siRNA (A) and dsRNA (B) injection reduces *Bm-cpl-1* transcript by 83% compared to controls (saline injected *Ae. aegypti* infected with *B. malayi*). *Bm-cpl-1* and control gene, *Bm-flp-14*, are normalized to a reference gene *Bm-thp-1*. qPCR was performed 48 h post-injection of RNAi trigger at 10 d post-infection. Each bar represents 13 mosquitoes from three biological replicates.

RT-qPCR was used to quantify the level of *Bm-cpl-1* suppression relative to two reference genes (*Bm-flp-14* and *Bm-tph-1*) using the efficiency-corrected (E^ΔΔCq^) relative quantification method [32]. PREXCEL-Q software was used to optimize the performance of the RT-qPCR assay; and important data pertinent to PCR efficiency, linear dynamic range and normalization of the assay are documented in Table 1. *Bm-tph-1* showed stable C_q_ values across the experiment and therefore was the most appropriate reference gene for these studies, as shown previously [33]. The suppressive effects of both RNAi treatments were almost identical; injection of 150 ng siRNA reduced *Bm-cpl-1* transcript by 83% compared to saline-injected controls (*P*<0.0001) and 150 ng dsRNA also reduced *Bm-cpl-1* transcript by 83% (*P*<0.0001) (Fig. 3). *Bm-flp-14* reference gene transcript was slightly reduced by both RNAi treatments but these reductions were not significant (siRNA, 9%, *P*  =  0.38; dsRNA, 12%, *P*  =  0.17). These data support the gel-based semi-quantitative RT-PCR experimental findings and demonstrate the efficacy of this novel method of RNAi delivery.

**Table 1:** Reportable information on RT-qPCR experiment.

	*Bm-cpl-1*		*Bm-flp-14*		*Bm-tph-1*	
	siRNA	dsRNA	siRNA	dsRNA	siRNA	dsRNA
RT-qPCR efficiency (%)	84.7	65	108.2	116.5	104.2	96.4
Calibration curve y intercept	30.6	32.9	33.9	35.9	29.5	33
Calibration curve r^2^	0.98	0.97	0.91	0.88	0.99	0.88
Mean C_q_	30.4±0.06	33.0±0.48	34.3±0.13	35.7±0.19	30.0±0.08	33.1±0.10
NTC C_q_	50	50	50	50	50	50

### *Bm-cpl-1* suppression elicits marked motility and developmental phenotypes

**Figure 4:**
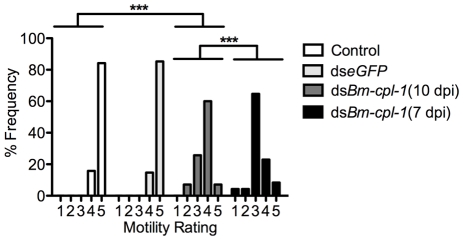
Aberrant motility of dsRNA *Bm-cpl-1*-exposed *B. malayi*. Frequency distribution for motility of L3 stage *B. malayi* recovered from *Ae. aegypti* showing significantly reduced motility of *Bm-cpl-1* suppressed worms. Parasitized mosquitoes were injected with saline (control), 150 ng *eGFP* dsRNA, or 150 ng *Bm-cpl-1* dsRNA at 7 or 10 d post-infection (dpi), then dissected to obtain parasites at 14 dpi. Parasite motility was scored on a 1–5 scale, with 1 =  immobile and 5 =  all parts of worm in constant motion (control n = 101, ds*eGFP* n = 68, ds*Bm-cpl-1* 10 dpi n = 70, ds*Bm-cpl-1* 7 dpi n = 48, *P*<0.001).

Previous studies have described aberrant filarial worm phenotypes associated with cathepsin L-like gene suppression *in vitro* including decreased microfilariae (mf) release from adult *B. malayi* [17] and an inhibition of the L3 to L4 molt in *Onchocerca volvulus* [18]. Based on these data, we predicted that *Bm-cpl-1* suppression would produce a phenotype *in vivo* in the mosquito host. Mosquitoes were injected with 150 ng *Bm-cpl-1* dsRNA 10 d post-infection (dpi) then microdissected four d post-injection to harvest L3-stage parasites. Worm motility was digitally recorded and scored according to a five-point schema of one (immobile), to five (all parts of the worm in constant motion). 100% of control worms from mosquitoes injected with *Aedes* physiologic saline were categorized as four or five on this scale. *Bm-cpl-1* suppression significantly inhibited this normal worm motility (*P*<0.001), with only 67% of worms ranked as four or five on the scale (Fig. 4). To confirm that this effect was *Bm-cpl-1* specific and not due to exogenous dsRNA impairing worm viability, this experiment was repeated with dsRNA for enhanced GFP (eGFP) as a random exogenous RNA. These worms were phenotypically identical to saline-injected controls (100% category four or five), confirming the specificity of the aberrant phenotype in *Bm-cpl-1* suppressed worms.

The effect of changing the timing of *Bm-cpl-1* suppression on worm motility was also examined. *Bm-cpl-1* transcript levels are elevated in L3 stage parasites, such that this gene has a purported role in the L3 to L4 molt [18]. The temporal expression of *Bm-cpl-1* was reported to be up-regulated during the L2 to L3 transition, at six to seven dpi [34]. Based on the timing of *Brugia* development in *Ae. aegypti* [35], infected mosquitoes were injected with *Bm-cpl-1* dsRNA at 10 dpi in order to target L3-stage worms (described above) and at seven dpi to target the L2 to L3 transition. Parasites exposed to *Bm-cpl-1* dsRNA at seven dpi showed significantly inhibited motility compared to saline controls (*P*<0.001) with only 31% of worms displaying normal motility. The difference between parasites exposed to dsRNA at seven and 10 dpi was significant (*P*<0.001), and may reflect an important biological role for *Bm-cpl-1* during the transition from L2 to L3 stages. More explicitly, earlier exposure to the RNAi trigger could impose more significant detrimental impact on the parasite by disrupting the L2 to L3 molt, or it may simply be a consequence of the longer period of time from gene suppression to phenotype assay, allowing Bm-CPL-1 rundown and maturation of the phenotype.

**Figure 5:**
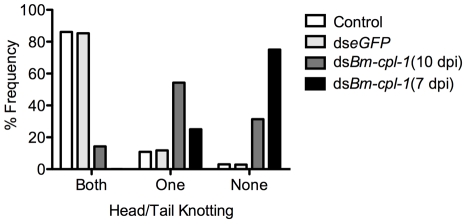
Disrupted motile phenotypes of dsRNA *Bm-cpl-1*-exposed *B. malayi*. Frequency distribution for parasites exhibiting rigorous knotting behavior at both ends of worm, one end, or not at all showing normal terminal curvature is inhibited by *Bm-cpl-1* suppression. Parasitized mosquitoes were injected with saline (control), 150 ng eGFP dsRNA, or 150 ng *Bm-cpl-1* dsRNA at 7 or 10 d post-infection (dpi), then dissected to obtain parasites at 14 dpi (control n = 101, ds*eGFP* n = 68, ds*Bm-cpl-1* 10 dpi n = 70, ds*Bm-cpl-1* 7 dpi n = 48).

**Figure 6:**
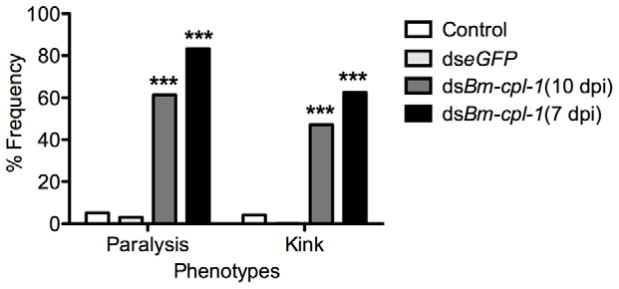
The frequency of caudal paralysis and kinked posture of dsRNA *Bm-cpl-1*-exposed *B. malayi*. The frequency of both caudal paralysis and kinked posture is significantly increased with *Bm-cpl-1* suppression. Parasitized mosquitoes were injected with saline (control), 150 ng *eGFP* dsRNA, or 150 ng *Bm-cpl-1* dsRNA at 7 or 10 d post-infection (dpi), then dissected to obtain parasites at 14 dpi (control n = 101, ds*eGFP* n = 68, ds*Bm-cpl-1* 10 dpi n = 70, ds*Bm-cpl-1* 7 dpi n = 48, *P*<0.001).

In addition to depressed activity, other morphological and motility phenotypes were apparent in *Bm-cpl-1* suppressed worms. A highly active, convoluted body form characterizes motility of healthy *B. malayi* L3s, both the heads and tails of the parasites in particular are conspicuously tortuous – curvature we described as ‘knotted’. Control worms from saline-injected mosquitoes frequently (86% of worms) displayed knotting at both ends. Suppression of *Bm-cpl-1* 10 dpi significantly inhibited this motility, because only 14% of worms presented with both ends knotted (*P*<0.001) (Fig. 5). This phenotype was enhanced by an early suppression of *Bm-cpl-1* at seven dpi such that no *Bm-cpl-*1 suppressed parasites exhibited this knotting morphology. The difference between L2 and L3 *Bm-cpl-1* suppression was significant (*P*  =  0.005). Worms exposed to the exogenous eGFP dsRNA control confirmed that this phenotype was gene-specific because parasite motility was not significantly different from saline controls (85% knotted at both ends, *P*  =  0.2). Another aberrant motility observed was the presence of a perturbed section of body wall slightly caudal to the midpoint of the worm. This abnormal kinked morphology was absent from control worms (4% of worms from saline-injected and 0% from eGFP-injected mosquitoes displayed this morphology), but evident with significantly greater frequency in 10 dpi *Bm-cpl-1* suppressed worms (47%, *P*<0.001) (Fig. 6). This kink rate increased with *Bm-cpl-1* suppression at seven dpi (63%), but compared to 10 dpi this was not significant (*P*  =  0.08). Finally, partial paralysis of *Bm-cpl-1* suppressed worms was evident, presenting as immobility in the caudal third of the worm. This paralysis was observed in 61% of 10 dpi *Bm-cpl-1* suppressed worms, and 83% of seven dpi suppressed worms (this increase was significant, *P*  =  0.005) but was generally absent from control worms (5% of worms from saline-injected mosquitoes and 3% of worms from eGFP-injected mosquitoes) (Fig. 6).

**Figure 7:**
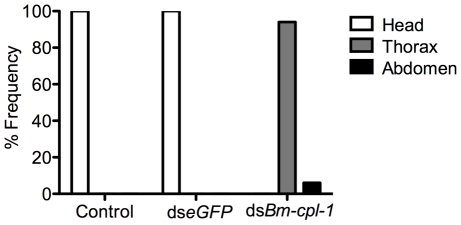
dsRNA *Bm-cpl-1*-exposed *B. malayi* fail to migrate to the head of the mosquito. Frequency distribution of infectious (L3) stage *B. malayi* recovered from *Ae. aegypti* in the head, thorax or abdomen of the mosquito host. Parasites were dissected from *Ae. aegypti* mosquitoes 14 d post-infection and 4 d post-injection of saline (control), 150 ng *eGFP* dsRNA or 150 ng *Bm-cpl-1* dsRNA. One worm was recovered from each mosquito. Numbers of mosquitoes dissected from three biological replicates: n = 18 (control), n = 20 (ds*eGFP*), and n = 31 (ds*Bm-cpl*-1).

To examine the consequence of this aberrant motility on *B. malayi* development, mosquitoes were injected with 150 ng *Bm-cpl-1* dsRNA 10 dpi then microdissected four d post-injection, partitioning the mosquitoes into head, thorax and abdomen preparations. Control worms from mosquitoes injected with either saline or eGFP dsRNA were found exclusively (100%) in head preparations as expected. *Bm-cpl-1* suppressed worms were most frequently observed escaping from the thorax and abdomen (Fig. 7). Parasites in *Bm-cpl-1* dsRNA-injected mosquitoes, however, did not leave the thorax (94% of worms were found here) or abdomen (6%). *Bm-cpl-1* suppression, therefore, prevents worm migration to the head of the mosquito, effectively preventing normal progression of the parasite life cycle and thus abolishing the potential for parasite transmission.

**Figure 8:**
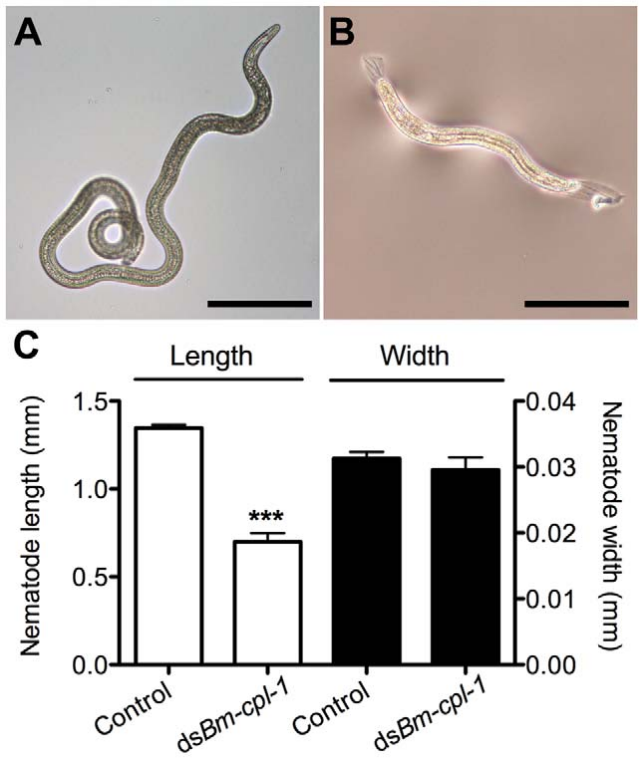
*Bm-cpl-1*-suppressed *B. malayi* are significantly shorter than control worms. Parasites were dissected from *Ae. aegypti* mosquitoes 14 days post-infection and 4 d post-injection of saline (control, A) or 150 ng *Bm-cpl-1* dsRNA (B). Scale bar 250 µm. (C) RNAi-exposed parasites are significantly shorter in length, but not width, than control worms. Numbers of parasites from three biological replicates: n = 19 (control) and n = 13 (ds*Bm-cpl*-1) (*P*<0.001).

A significant negative effect also was seen on growth and development of parasites subjected to *Bm-cpl-1* suppression. Parasitized mosquitoes were injected with 150 ng *Bm-cpl-1* dsRNA seven dpi then microdissected 14 dpi and the length, width and appearance of the worms recorded. Mosquitoes were injected seven dpi because the previous motility experiments dictated that this experimental timing generated the most pronounced motility phenotypes. *Bm-cpl-1* suppression significantly reduced the length of L3 worms by 48% (*P*<0.0001)(Fig. 8). The mean length of control L3, removed from mosquitoes seven d after saline injection and 14 dpi, was 1347±18 µm. This was reduced to 700±49 µm after RNAi treatment. Unlike parasite length, width was not significantly affected by *Bm-cpl-1* suppression, although a slight decrease of 5% was observed (*P*  =  0.39) from 31±1 µm in control worms to 30±2 µm in RNAi worms. In addition to worm length, the majority of *Bm-cpl-1* dsRNA parasites also presented with additional aberrant developmental phenotypes. Most evident was a disruption of the cuticle (Fig. 8B), which extended significantly beyond the body of the worm. Some degree of this cuticular sloughing was noticed in most worms but the severity of this phenotype was variable. Finally, the integrity of the gut appeared compromised in *Bm-cpl*-1 worms. In such instances, the gastrointestinal tract of the parasites appeared incomplete and porous when examined at the light microscope level.

### *Bm-cpl-1* suppression enhances mosquito survival and decreases parasite prevalence

**Figure 9:**
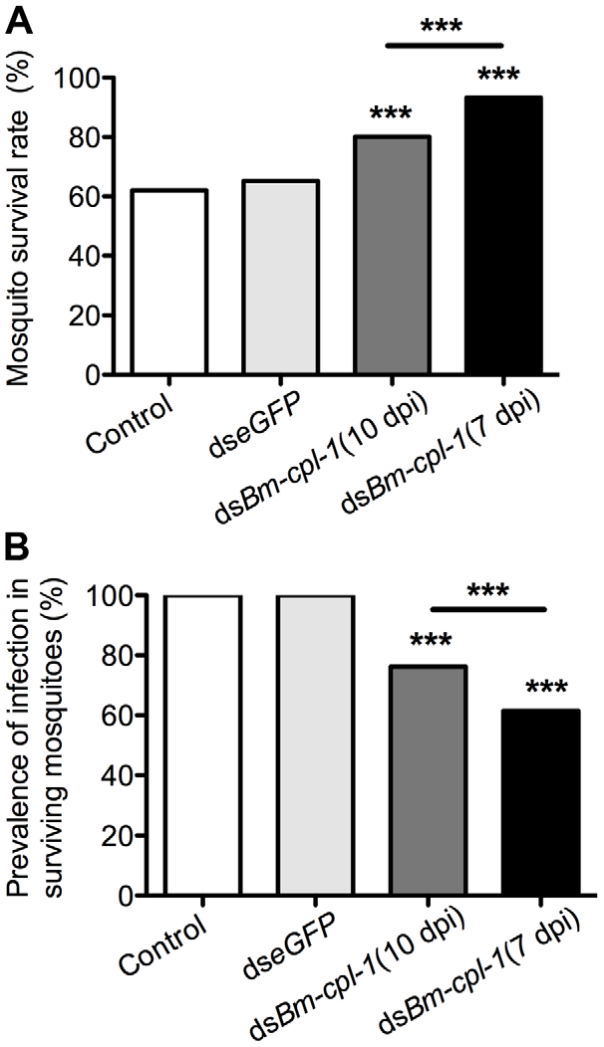
*Ae. aegypti* survival significantly increases as infection prevalence decreases in *Bm-cpl-1*-dsRNA exposed mosquitoes. The effects on survival (A) and infection prevalence (B) are most profound in parasites exposed to *Bm-cpl-1* dsRNA at the L2/L3 transition (7 d post-infection, dpi). Parasites were exposed to saline (control), 150 ng *eGFP* dsRNA or 150 ng *Bm-cpl-1* dsRNA at 7 or 10 dpi, then were dissected from *Ae. aegypti* mosquitoes at 14 dpi.

Phenotype data resoundingly demonstrate that *Bm-cpl-1* suppression decreases *B. malayi* viability. It is logical to predict that this decreased viability would also have an impact on mosquito survival and prevalence of parasite infection. To examine this, mosquitoes were injected with 150 ng *Bm-cpl-1* dsRNA 10 dpi and the number of mosquitoes that survived through the development of parasites to the infectious stage, 14 dpi, was counted. Surviving mosquitoes then were microdissected to determine the proportion that harbored parasite infections. *Bm-cpl-1* suppression increases host mosquito survival. The survival rate of control mosquitoes injected with saline or eGFP dsRNA was 62% and 65% respectively (*P*  =  0.6) as compared to 80% in *Bm-cpl-1* RNAi-exposed mosquitoes (*P*<0.001) (Fig. 9A). Early suppression of *Bm-cpl-1* at seven dpi enhanced the phenotype even more significantly such that 93% of mosquitoes were alive at the termination of the experiment (*P*  =  0.007). This increased mosquito survival rate after *Bm-cpl-1* suppression may be as a result of the parasite's compromised ability to feed on, and migrate through, the host or may result from a more successful or effective host response against parasites with decreased viability. This hypothesis is supported by our observation that *Bm-cpl-1* suppression also decreased prevalence of infection – fewer surviving mosquitoes harbored parasites after *Bm-cpl-1* RNAi (Fig. 9B). Every surviving mosquito injected with saline or eGFP was found to contain parasites 14 dpi, but 14 dpi *Bm-cpl-1* suppressed parasites (exposed to dsRNA at 10 dpi) were found in just 76% of mosquitoes, a significant reduction in prevalence (*P*<0.001). Prevalence was further reduced to 62% in parasites exposed to *Bm-cpl-1* dsRNA at seven dpi, a statistically significant decrease compared to worms exposed to the dsRNA trigger at 10 dpi (*P*  =  0.03).

## Discussion

Here we report the development of a novel *in vivo* approach to RNAi in the filarial nematode *Brugia malayi*, and describe its application first to suppress the expression of *Bm-cpl-1*, a *B. malayi* gene encoding a cathepsin L-like cysteine protease, then to validate this gene as a potentially potent anthelmintic drug target. To the best of our knowledge, this is the first description of *in vivo* RNAi in parasitic nematodes and represents an advance in the study of filarial nematode biology that may aid in the development of drugs to combat parasitic nematode infection. The rationale for developing an *in vivo* RNAi protocol stems from the hypothesis that RNAi is ineffective in animal parasitic nematodes because the supply of an RNAi trigger to the worms is inappropriate [29]. Our overarching hypothesis was that RNAi would work effectively and robustly if a trigger is supplied to healthy, viable worms in a host environment. Supporting this hypothesis, we were able to specifically reduce target gene transcript abundance in *B. malayi* larvae by 83% by supplying an RNAi trigger to parasites developing within the mosquito host. This level of transcript knockdown has not previously been reported using current *in vitro* RNAi soaking methods. The ‘*in squito’* approach to RNAi we describe is effective for the specific suppression of cathepsin genes in *Brugia* larval stages as they develop within their cognate mosquito host; it is therefore possible that this *in vivo* approach may represent a more effective means of eliciting gene suppression in filarial nematodes.

The mechanism by which the RNAi trigger is delivered to the parasites ‘*in squito’* is unclear, but could be a result of bathing the parasite in the trigger within a cell, or as a result of uptake by tissue ingestion. In support of the former, Cy3-labeled siRNA injected into the haemocoel rapidly disseminates throughout the mosquito supporting a hypothesis that the developing parasites are effectively incubating in a host milieu containing an RNAi trigger, essentially a scenario analogous to *in vitro* RNAi by soaking. If this is the case, the ‘*in squito*’ approach represents an efficient way to generate gene suppression by soaking. Most successful animal parasitic nematode *in vitro* soaking protocols use large amounts of ds- or siRNA with concentrations of 1 mg/ml typical, meaning anywhere between 25 µg and 2 mg of RNAi trigger are required per suppression experiment [10]–[19], with the exception of one report showing that lower trigger concentrations could still be effective at producing gene suppression by soaking [20]. Here we showed that gene suppression can be achieved using just 150 ng of ds- or siRNA per RNAi event, and indeed, a reduction in transcript abundance was observed after injecting as little as 15 ng dsRNA. In addition to the obvious cost saving advantages to performing RNAi experiments in this manner, such low RNA concentrations may also improve the specificity of gene suppression. Soaking plant parasitic nematodes in serial dilutions of ds- and siRNAs has been shown to reduce off-target effects in RNAi experiments [28], [36]. A second delivery hypothesis is that the developing parasites are ingesting the RNAi trigger. Microfilariae taken in during the blood meal rapidly penetrate the mosquito midgut [37], and migrate to the thoracic musculature where they grow and develop to the L3 stage [38], [39], a process completed in under two weeks [35]. From the L2 stage, the developing larvae are active feeders and consume host tissue [35], [40], [41], a behavior that would lead to the ingestion of an injected RNAi trigger in our experimental model. RNAi by feeding is a well-established method in free-living nematodes [22], [23], [42], [43]; by feeding these worms bacteria expressing dsRNA, systemic gene suppression can be effected in a relatively simple and efficient manner. This approach has not been successful with parasitic nematodes, however, as most parasitic species are not bacteriotrophic, and even for those species with bacteriotrophic life stages, this method is unreliable [30]. Resolution of the RNAi trigger delivery mechanism afforded by our *in vivo* protocol may come through targeting *B. malayi* L1 worms in the mosquito. If target gene expression can be reduced in this non-feeding stage, this would support soaking as the prime mechanism.

The *in squito* suppression of *Bm-cpl-1* reveals new phenotypes associated with molting, growth and development, and motility that shed light on the important biological functions of this gene family in larval stages of *B. malayi.* Nematode molting is a three-stage process characterized by a shedding or separation of the old cuticle from the epidermis (apolysis), generation of a new cuticle, then the shedding of the old cuticle (ecdysis). The use of specific cysteine protease inhibitors markedly inhibits the L3 to L4 molt in filarial worms implicating cysteine proteases in general in this process [34], [44], [45]. More explicitly, both apolysis and ecdysis are disrupted giving rise to L4 parasites constrained within an L3 cuticle, termed an ‘accordion’ phenotype [34]. Multiple members of the cathepsin L-like family appear to be involved in molting as the specific suppression of *cpl-1* alone in *Onchocerca vovlvulus* reduced but did not abrogate the L3 to L4 molt [18]. We show that *Bm-cpl-1* is also involved in similar processes in *B. malayi* as its suppression manifested an aberrant cuticular phenotype in L3 worms. Examination of worms suppressed at seven dpi revealed an apparent sloughing of the cuticle without the accordion phenotype. As the L3 to L4 molt occurs in the vertebrate host this phenotype is not a disruption of the L3 to L4 molt, but rather a dysfunction in L3 cuticle formation, maintenance or development. Alternatively, we could be observing a disruption of the L2 to L3 molt. *Bm-cpl-1* expression is up-regulated in the L3 stage but the exact timing of this up-regulation as it relates to the transition from L2 to L3 stages is unclear. Guiliano et al. [34] report *Bm-cpl-1* up-regulation at six dpi, a window consistent with the L2 to L3 transition. If *Bm-cpl-1* performs the same function for the L2 to L3 molt as for the L3 to L4 molt, then the sloughed cuticle we observe upon dsRNA injection at seven dpi could be that of the L2, with *Bm-cpl-1* suppression preventing ecdysis. Further examination of cuticle ultrastructure in these suppressed worms at the electron microscope level could provide evidence to this effect.

We observed a stunting of *Bm-cpl-1* suppressed L3 growth compared to control worms, a phenotype previously unreported either after chemical inhibition of cysteine proteases or gene suppression in other parasite stages. Normally at the end of the L2 stage parasites are 750–795 µm long and increase in length to approximately 1350 µm at the L3 stage within four d [38]. Our control L3, taken from mosquitoes injected with saline, had a mean length of 1347 µm corresponding closely with the published data. The mean length of *Bm-cpl-1* suppressed L3, however, was significantly shorter (700 µm). Suppression of this gene at the L2/L3 interface (seven dpi) arrests parasite growth and the L3 worms remain L2-sized within the mosquito. One explanation for this observation is that *Bm-cpl-1* suppression at the L2/L3 interface is inhibiting the L2 to L3 molt, L2 cuticle ecdysis is not successful and therefore the worms are constrained within it, unable to increase their length. Alternatively, the stunting may not be due to aberrant molting but rather an inhibition of normal CPL-regulated development or cellular remodeling post-molt as is seen in other nematodes [46]. RNAi suppression of *cpl-1* in *C. elegans* L3 by soaking produced significantly shorter and thinner adults [46] and the localization of *cpl-1* to the hypodermis in *C. elegans*, *O. volvulus* and *B. malayi* is consistent with a developmental role in nematodes [18], [34], [46]. Further, germline suppression of *cpl-1* in *C. elegans* by dsRNA injection generated an embryonic lethal phenotype but some embryos did progress to the L1 stage and those had incomplete gut development [46]. A repeatedly observed phenotype in our *Bm-cpl-1* suppressed L3 was a compromised gut that appeared fenestrated and poorly developed.

Finally, *Bm-cpl-1* suppression reduced normal L3 motility by up to 69%, increased atypical postural phenotypes including caudal paralysis, kinked appearance and reduced normal convolution at the head and tail of *B. malayi* L3 as compared to control worms. These behaviors made it impossible for the treated L3 to progress through to the culmination of development in the mosquito host, i.e., transfer to the definitive host. The dystaxic behaviors produced by the suppression of *Bm-cpl-1* suggest this gene has some role, directly or indirectly, in the neuromuscular activity of *B. malayi* L3 in the mosquito. It is certainly true that cathepsins are required for normal neuromuscular behavior in other helminths; suppression of a cathepsin L-like gene in the flatworm *Fasciola hepatica* generated several aberrant motile phenotypes including paralysis [47].

This study is the first to use the host as a delivery mechanism for animal parasitic nematode RNAi. The model of using the host as a delivery mechanism for RNAi has been established but has been restricted to plant pathology where the concept has an applied use with transgenic plants helping to control nematode infestation by RNAi mechanisms *in planta* [48]–[50]. An alluring corollary is that by generating transgenic mosquitoes capable of suppressing key nematode genes *in vivo* we may be able to abolish parasite transmission. We have already demonstrated here that *Bm-cpl-1* suppression *in vivo* prevents parasites migrating to the mosquito head and proboscis thus eliminating transmission potential. Transformation of a mosquito with an inverted-repeat (IR) transgene derived from *Bm-cpl-1* may result in endogenous transcription of a hairpin dsRNA, a trigger that conceptually would induce RNAi *in vivo* as described here and produce a mosquito incapable of transmitting lymphatic filariasis-causing worms. Methods to introduce transgenes into mosquito germlines are well established [51]–[55] and proof of this principle has already been demonstrated for a mosquito-borne virus; transgenic lines of Dengue virus-resistant mosquitoes were generated using a Dengue virus IR transgene driven by the carboxypeptidase A promoter, reducing virus transmission by an RNAi mechanism [56]. The viability of this approach is enhanced not only by the ability to transform important vector species but also by the identification of tissue-specific promoters to drive transgene expression in favorable tissues, for example, *act88F* [57] is a fly-specific promoter that drives gene expression in the flight musculature – the precise site of parasite development. Another positive impact this protocol may have on lymphatic filariasis control is as a means of better understanding the biology of current putative drug targets and generating new data that may validate proposed novel drug targets. This protocol introduces the ability to investigate mosquito-borne parasite life stages, allowing the critical examination of gene function in worms growing and developing in an optimum environment. This makes it possible to assay genes that encode known or proposed drug targets in a parasite within its native intermediate host, contextualizing the null phenotypes *in vivo* and accurately determining the consequences of target gene suppression producing a more valuable target validation. As an illustration, nematode cathepsins have been proposed as attractive novel drug targets [58] and we have further validated these drug targets *in vivo*, revealing new phenotypes, defining new biological roles and showing that *B. malayi sans Bm-cpl-1* are incapable of completing their life cycle. These data enhance the appeal of cathepsins as novel anthelmintic drug targets. Beyond cathepsins, this technique will have most utility in the investigation of known and potential antifilarial drug target genes expressed in both the mosquito-borne life stages and those life stages that are vulnerable to chemotherapeutic intervention.

In summary, we have developed an innovative RNAi protocol using *B. malayi* that differs conceptually from present RNAi protocols in that parasite gene expression is suppressed within the mosquito intermediate host. Using this protocol we suppressed a *B. malayi* gene *in vivo*, eliciting aberrant developmental and motility phenotypes in the parasite – phenotypes that eliminate transmission potential. In contrast to present RNAi methods, we have found the protocol to be reliable and effective, providing a major advancement in our capability to better understand filarial nematode gene function to the benefit of human health.

## Materials and Methods

### Mosquito maintenance and injection protocol

*Aedes aegypti* (Liverpool strain), previously selected for susceptibility to filarial parasites [59], were maintained in a contained environment at a constant temperature of 25°C, 80% relative humidity and a 14 h light to 10 h dark photoperiod. The mosquitoes were fed a diet of 0.3 M sucrose. Throughout the study mosquitoes to be injected were anaesthetized on ice and immobilized on a vacuum saddle before being microinjected intrathoracically at the base of the head using a pulled borosilicate glass pipette attached to a manual syringe for injection by volume displacement. A maximum volume of 0.5 µL can be injected using this approach with a high mosquito survival rate (>95%).

### Establishing *Brugia* infection

*B. malayi* microfilaria (mf) infected cat blood was obtained from the University of Georgia NIH/NIAID Filariasis Research Reagent Resource Center. To establish a consistent and repeatable parasitemia, mf were first purified using a filtration protocol [60]. Blood containing the parasites was diluted with phosphate buffered saline (1∶5 ratio, blood:PBS) then syringe filtered through a 0.45 µm Millipore filter. Captured mf were washed three to five times with PBS then a further three to five times with *Aedes* physiologic saline [61] before centrifugation at 6,800× g for five min. Supernatant was removed and the pelleted mf resuspended in fresh *Aedes* saline to a concentration of 40 worms per µL. To inoculate mosquitoes, 20 mf were injected as described. Microdissection of the mosquitoes throughout a 14 dpi period confirmed this method established a controlled infection that progressed in a predictable and consistent manner. We also tried a blood feeding approach to establish infection but this produced an inconsistent worm burden that is too variable to reliably assess subsequent gene suppression experiments.

### siRNA and dsRNA generation and injection

Short interfering RNAs (siRNA) targeting a *B. malayi* cathepsin L-like gene (*Bm-cpl-1* AF331035 [34]) were generated commercially (Qiagen, CA) and modified with a 3′-Cy3 fluorophore on the sense strand. The location of each siRNA was optimized using a proprietary algorithm and the sequence of each siRNA is as follows: BmCL1-1, AAGGCTTAGTTTCTTATACAA; BmCL1-2, CCGAATGGAAAGATTATGTAA; BmCL1-3, CAGAAGTGCATTGAAGGAATA; and BmCL1-4, CCGGTATTTACTCCAGTAATA. Equimolar amounts of each siRNA were combined and this mix was used for injection and gene suppression experiments. dsRNA duplexes were generated in-house using a T7 transcription-based approach. A 410 base pair transcription template was polymerase chain reaction (PCR) amplified from a *B. malayi* L3 stage cDNA library (kindly provided by Dr. S. Williams, Smith College, MA) using gene specific oligonucleotides designed to incorporate a T7 promoter sequence (TAATACGACTCACTATAGGGTACT) at both the 5′ and 3′ ends of the amplicon. For the *Bm-cpl-1* template, oligonucleotide sequence was: L1T7dsRNAF 5′ TAATACGACTCACTATAGGGTACTACGGTTACCAAATTC 3′ and L1T7dsRNAR 5′ TAATACGACTCACTATAGGGTACTCGACAACAACAGGTC 3′. The location of this transcription template was carefully chosen so as to exclude the pro region of *Bm-cpl-1*, a domain with high sequence homology to other cathepsin L family genes, and consequently increase the specificity of this dsRNA duplex. Transcription templates were gel purified and dsRNA duplexes synthesized using the MEGAscript RNAi Kit (Ambion, TX) according to manufacturer's protocols. dsRNA species were quantified with a NanoVue spectrophotometer (GE Healthcare, NJ) prior to use. The timing of siRNA or dsRNA injection into *B. malayi*-infected mosquitoes coincided with the presence of the parasite stage of interest: to target second larval stage (L2) parasites siRNA or dsRNA were injected five to eight dpi; to target third larval stage (L3) parasites siRNA or dsRNA were injected nine to 12 dpi (and for the lifespan of mosquito) [35]. The mosquitoes were processed to confirm suppression of the target gene, as described below, 48 h post-injection of siRNA or dsRNA.

### Relative quantitative RT-PCR

*Brugia* infected, RNA-treated and control mosquitoes were cold-anesthetized on ice. Total RNA was extracted from individual mosquitoes using RNAqueous Kit (Ambion, TX) before DNase treatment using the TURBO DNA-free Kit (Ambion, TX) in thin-walled PCR tubes. The RNA was stabilized with RNase Out Inhibitor (Invitrogen, CA) and stored in RNase-free microcentrifuge tubes at 4°C. This RNA was used as a template for a relative semi-quantitative multiplex RT-PCR using the SuperScript III One-Step RT-PCR System with Platinum Taq DNA Polymerase (Invitrogen, CA). The principle of this reaction is to amplify a target gene of interest and compare its intensity with a multiplexed and normalized internal standard during the linear phase of product amplification. A putative neuropeptide encoding gene, *Bm-flp-14* (Accession number AI508026) served this role. This gene was chosen as we had previously determined its stable transcript production during the *B. malayi* L3 stage by PCR (C. Song, unpublished). The oligonucleotide primers used to amplify *Bm-cpl-1* were: CPL-1 F 5′ ACAGGGCAATATGACGAGAC 3′ and CPL-1 R 5′ ATCGAAGCAACGTGGCACAT 3′. These primer locations flank the region of *Bm-cpl-1* homologous to the dsRNA construct. The oligonucleotide primers used to amplify the *Bm-flp-14* internal standard were: FLP-14 F 5′ CTCGTCCACTCTTATCACTG 3′ and FLP-14 R 5′ ACCGCAATGATATACAACATATA 3′. The profile for this PCR was: cDNA synthesis at 50°C for 30 minutes; an initial denaturation phase of 94°C for 2 min; 38 cycles of 94°C for 30 s, 60°C for 30 s, 68°C for 1 min and a final extension phase of 68°C for 5 min. Reactions were visualized on a 1.2% agarose gel containing ethidium bromide.

### Quantitative RT-PCR

Total RNA was extracted from individual mosquitoes and DNase-treated as described above for three replicated RNAi experiments and before addition of RNase Out Inhibitor and storage, each RNA sample was quantified spectrophometrically per a previous report [62]. This RNA served as a template in our RT-qPCR assays using the qScript One-Step Fast RT-PCR Kit with ROX (Quanta BioSciences, MD).

#### Establishing PREXCEL-Q parameters

PREXCEL-Q, a qPCR assay development and project management software, was used to establish our RT-qPCR parameters and to determine valid working ranges for all of our samples per target and reference genes. A mixture of the RNA samples was used to determine the optimum template to use while avoiding RT-qPCR inhibition for each of the three targets at concentration ranges of between 0.01 and 0.08 ng/µL for the siRNA experiments and between 0.02 to 0.14 ng/µL for the dsRNA experiments. For subsequent quantitative assessment of transcript abundance, each RNA sample was diluted to 0.06 ng/µL for the siRNA RT-qPCR study and 0.11 ng/µL for the dsRNA RT-qPCR study, with 6 µL of sample used per 25 µL reaction.

#### Primers and probes

The target sequence under evaluation in the RT-qPCR study was the *B. malayi* cathepsin L-like transcript previously described. Two reference genes were used, a neuropeptide-encoding gene *Bm-flp-14* also previously described, and *Bm-tph-1* (Accession number U80971), a tumor protein homolog-encoding gene that is a proven reference gene for qPCR of *Brugia* development in mosquitoes [33]. TaqMan minor groove binding (MGB) probes were used in this study to facilitate the use of shorter gene specific primer-probe sets. All probes and primers were designed using Primer Express v. 2.0 software (Applied Biosystems, CA) and synthesized by Applied Biosystems. The primer and probe sequences used are shown in Table 2.

**Table 2:** Primer and TaqMan probe sequences for RT-qPCR experiment.

Gene	Primer sequence (5′ - 3′)		Amplicon Size (bp)
*Bm-cpl-1*	ForwardReverseProbe	GGTTACGGAACGCATCGAA TGGGTTCCCCAGCTATTTTTAA6FAM-TCACGGTGATTACTGGAT-MGBNFQ	62
*Bm-flp-14*	ForwardReverseProbe	TGGGAAGAGGAAGCATGAATACTT TGCAGCGGGAACTTTGATC6FAM-AGATTTGGTCGTAAGTAGTTG-MGBNFQ	66
*Bm-tph-1*	ForwardReverseProbe	TTGCAACGATATGTTGATCTTCAA ACGAGTCCGACGCAAGCT6FAM-ATGCATTCACAGATGAC-MGBNFQ	62

#### TaqMan RT-qPCR

25 µL volume reactions were prepared in duplicate for each RNA sample, and 20 µL of this reaction mixture is applied per well on a 96-well plate (using white-well reaction plates, Eppendorf, NY). Individual components of each RT-qPCR reaction were as follows: 6 µL prediluted RNA (as determined by PREXCEL-Q), 6.25 µL 4X One-step Fast Master Mix with ROX, 1.25 µL qScript One-Step Fast RT, 775 nM each primer, 150 nM probe, nuclease-free water to 25 µL. Cycling conditions included an initial cDNA synthesis step of 50°C for 5 min followed by an RT denaturation/Taq activation phase of 95°C for 30 s then 45 cycles of 95°C for 3 s and 58°C for 30 s. Four point standard curves were created for each target (within the ng/µL ranges already specified above) by diluting the RNA sample mixture in each case according to precise, PREXCEL-Q-determined parameters (eight-fold dilution from highest to lowest concentration). No-template control reactions substituted nuclease-free water for RNA, and thermocycling was performed on an ABI GeneAmp 5700 SDS (Applied Biosystems). Quantification cycle (C_q_) values were obtained at an appropriate threshold per each target (∼0.1 DR_n_ in all cases), and data were processed using custom Excel files by the efficiency-corrected (E^ΔΔCq^) relative quantification method [32].

### Phenotype analysis

After confirmation of *Bm-cpl-1* suppression, multiple assays were performed to describe worm phenotypes. Each phenotypic assay was performed 14 dpi and at either four or seven d post-injection. Mosquitoes were cold-anesthetized then the wings and legs removed and discarded using a dissecting microscope. The head, thorax and abdomen were partitioned and further dissected to release the parasites. The following characteristics of dissected parasites were observed: (1) *Parasite location*. In order to be successfully transmitted, these parasites have to actively migrate to the head of the mosquito and vigorously writhe free of the proboscis. Parasite migration through the mosquito was recorded and measured according to escape point from the mosquito body (abdomen, thorax or head). (2) *Worm motility*. A scoring schema of: one (immobile), two (compromised motility, immobile for stretches of time), three (sluggish, partial movement), four (in motion, some straight segments), or five (all parts of the worm in constant motion) was used to quantify parasite movement in a blind fashion by an independent evaluator. Additional observations of aberrant motility included knotting at one or both ends, paralysis of caudal region and presence of a distinct angular kink were also recorded. (3*) Parasite growth and development*. Digital images of RNAi and control worms were captured so that length and diameter could be calculated using NIS Elements D 2.30 software (Nikon, NY). (4) *Parasite viability*. The number of parasites that survived to the infectious stage was recorded so that infection prevalence and mean intensity could be calculated. (5) *Mosquito viability*. We documented the number of mosquitoes that survived through the development of parasites to the infectious stage because these parasites inflict significant pathology and decrease mosquito survival.

### Microscopy

Nikon Eclipse *50i* fluorescence microscope under UV light (EXFO, ON) equipped with a Hy-Q FITC filter set (Chroma, VT). Images were captured using a Digital Sight DS-2Mv camera and NIS Elements D 2.3 software (Nikon, NY).

### Statistical analysis

*t*-tests were used to analyze the effect of RNAi treatment on gene expression in the RT-qPCR experiments and parasite size, and ANOVA to analyze the effect of RNAi treatment on worm motility based on our one through five blind-scoring schema. Chi square tests were used to analyze the effect of RNAi treatment on all other worm and mosquito behaviors assayed. In all tests, *P* values ≤0.05 were considered statistically significant.
